# Quantified CIN Score From Cell-free DNA as a Novel Noninvasive Predictor of Survival in Patients With Spinal Metastasis

**DOI:** 10.3389/fcell.2021.767340

**Published:** 2021-12-09

**Authors:** Su Chen, Minglei Yang, Nanzhe Zhong, Dong Yu, Jiao Jian, Dongjie Jiang, Yasong Xiao, Wei Wei, Tianzhen Wang, Yan Lou, Zhenhua Zhou, Wei Xu, Wan Wan, Zhipeng Wu, Haifeng Wei, Tielong Liu, Jian Zhao, Xinghai Yang, Jianru Xiao

**Affiliations:** ^1^ Department of Orthopedic Oncology, Changzheng Hospital, Naval Medical University, Shanghai, China; ^2^ Center of Translational Medicine, Naval Medical University, Shanghai, China; ^3^ Weizmann Institute of Sciences, Rehovot, Israel

**Keywords:** tokuhashi score, prognosis, CIN score, CNV, spine metastasis

## Abstract

**Purpose:** Most currently available scores for survival prediction of patients with bone metastasis lack accuracy. In this study, we present a novel quantified CIN (Chromosome Instability) score modeled from cfDNA copy number variation (CNV) for survival prediction.

**Experimental Design:** Plasma samples collected from 67 patients with bone metastases from 11 different cancer types between November 2015 and May 2016 were sent through low-coverage whole genome sequencing followed by CIN computation to make a correlation analysis between the CIN score and survival prognosis. The results were validated in an independent cohort of 213 patients.

**Results:** During the median follow-up period of 598 (95% CI 364–832) days until December 25, 2018, 124 (44.3%) of the total 280 patients died. Analysis of the discovery dataset showed that CIN score = 12 was the optimal CIN cutoff. Validation dataset showed that CIN was elevated (score ≥12) in 87 (40.8%) patients, including 5 (5.75%) with head and neck cancer, 11 (12.6%) with liver and gallbladder cancer, 11 (12.6%) with cancer from unidentified sites, 21 (24.1%) with lung cancer, 7 (8.05%) with breast cancer, 4 (4.60%) with thyroid cancer, 6 (6.90%) with colorectal cancer, 4 (4.60%) with kidney cancer, 2 (2.30%) with prostate cancer, and 16 (18.4%) with other types of cancer. Further analysis showed that patients with elevated CIN were associated with worse survival (*p* < 0.001). For patients with low Tokuhashi score (≤8) who had predictive survival of less than 6 months, the CIN score was able to distinguish patients with a median overall survival (OS) of 443 days (95% CI 301–585) from those with a median OS of 258 days (95% CI 184–332).

**Conclusion:** CNV examination in bone metastatic cancer from cfDNA is superior to the traditional predictive model in that it provides a noninvasive and objective method of monitoring the survival of patients with spine metastasis.

## Introduction

The skeletal system is the third most common metastatic site of most cancers (Sutcliffe et al., 2013). Postmortem examination showed that about 70% of cancer patients had spinal metastases (Bartels et al., 2008). Intractable pain, neurologic deficits, and paralysis are common symptoms of these patients, fundamentally impacting their survival and quality of life (Tang et al., 2016). Survival time of the patients with bone metastases is a key consideration for the decision-making of subsequent treatments. However, there is a lack of robust clinical tools or biomarkers to accurately predict the prognosis of bone metastatic patients.

Several scoring systems including Tomita, Sioutos, and Van der Linden scores have been developed (Tomita et al., 2001; Sioutos et al., 1995; van der Linden et al., 2005), and the revised Tokuhashi score is the most commonly used in survival prediction of cancer patients (Tokuhashi et al., 1990; Tokuhashi et al., 2005). However, previous studies have demonstrated that the revised Tokuhashi score is not accurate enough in prognostic prediction of patients with spinal metastasis, and therefore the survival of patients with low a Tokuhashi score (≤8) might be underestimated (Tan et al., 2016; Yang et al., 2019). Precise selection of these patients could improve therapeutic planning and ameliorate the clinical outcomes.

The implementation of molecular diagnostics involves scalability, sensitivity, and specificity. In patients with spinal metastasis, whole-genome sequencing analysis of cell-free DNA has emerged to be a promising strategy for reflecting the burden and genetic alterations of tumors (Ellinger et al., 2011). The profiling of genetic alterations in circulating cell-free DNA from patient plasma has potential clinical applications including early disease detection, real-time prediction of treatment response, and prognostication (Tran et al., 2021). In various cancer types, cfDNA unveils abundant information including the metastatic status, microsatellite instability, somatic copy number alterations, and the structural rearrangements (Vanderstichele et al., 2017; Mayrhofer et al., 2018). Copy number variation (CNV) is the result of ongoing changes in the number or structure of chromosomes, which was a hallmark in various cancers (Vanderstichele et al., 2017; Gronroos and López-García, 2018). Initially, maternal neoplasia was incidentally detected by identifying chromosomal and/or subchromosomal abnormalities during noninvasive prenatal testing (Bianchi et al., 2015; Dharajiya et al., 2018). A later study on the large population validated a novel cancer detection pipeline algorithm to enhance the detection of occult maternal malignancies through identifying multiple chromosomal aneuploidies (Ji et al., 2019). In addition to noninvasive prenatal testing, other noninvasive examinations focusing on chromosomal instability analysis suggested high CNV detected in cfDNA could serve as surrogates for predicting and monitoring malignancies (Oellerich et al., 2017).

In this study, low-coverage whole-genome sequencing of cfDNA was conducted to examine blood plasma from patients with spinal metastasis. An analysis pipeline was developed and validated to evaluate the CNV status in cfDNA, in an attempt to determine whether the CIN score, which was defined as a prognostic factor in patients with metastatic cancer like breast cancer (Mo et al., 2020), could also predict the survival of pan-cancer patients with spinal metastasis and especially identify the patients with actual good prognosis but ranked with a low Tokuhashi score.

### Patients and Methods

#### Patients and Study Design

**FIGURE 1:**
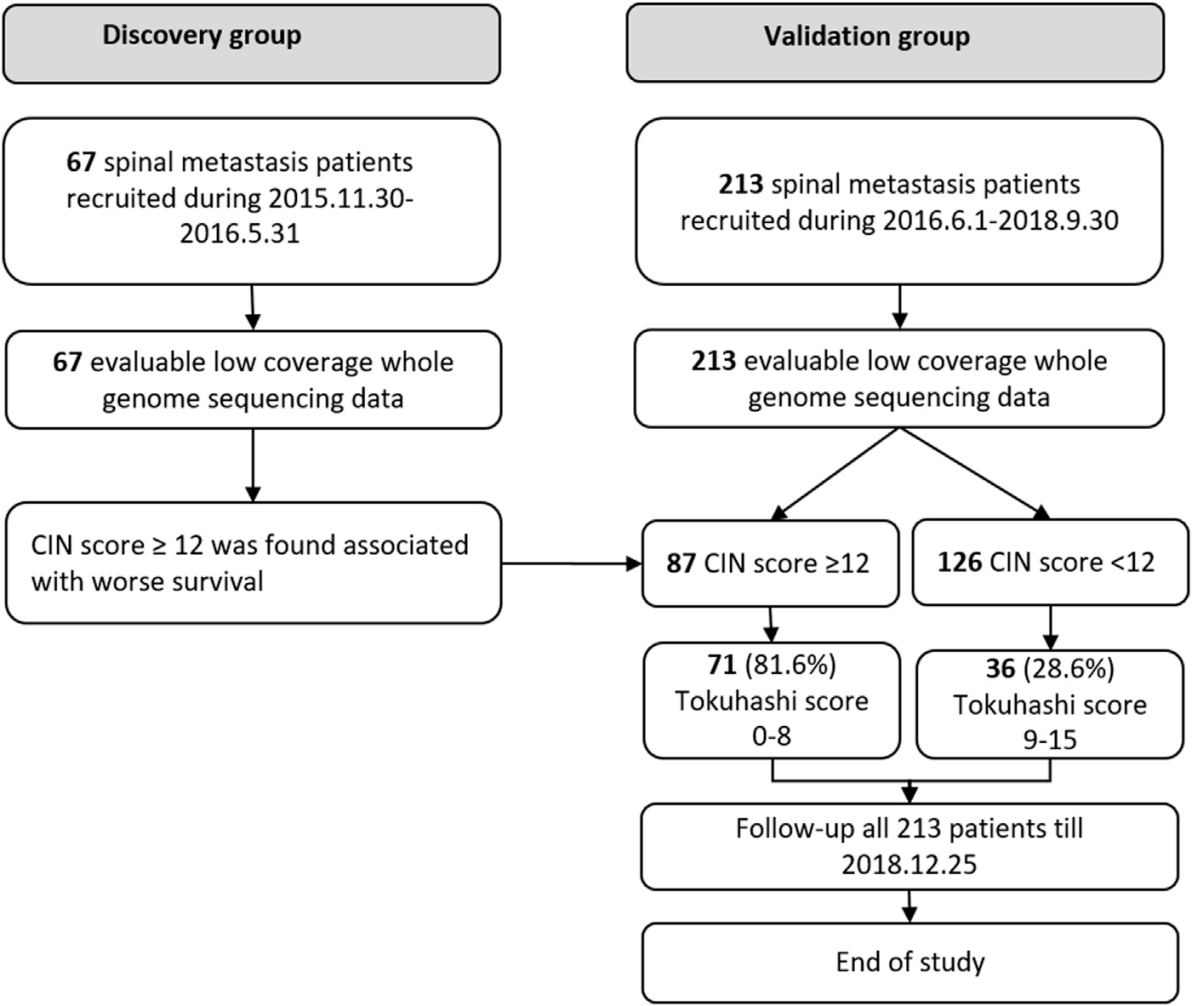
Study design.

This cohort study was approved by the institutional review board. Written informed consent was obtained from all participating patients. Between November 2015 and May 2016, 67 patients with spinal metastasis were retrospectively included in the discovery group, and between June 2016 and September 2018, 213 patients were included as a prospective cohort for final validation (validation group), shown in Figure 1. The inclusion criteria are as follows: patients diagnosed with radiologically and pathologically confirmed spinal metastases. Blood samples were collected in EDTA anticoagulant tubes (BD, USA) before the surgical treatment and centrifuged to obtain plasma within 40 min. Patients with unqualified blood samples were excluded. All the patients were preoperatively assessed according to the revised Tokuhashi score (Tokuhashi et al., 2005). Clinical data were abstracted from the medical records, including the general condition, primary tumor site, visceral metastasis, vertebral metastasis, extraspinal metastasis, and neurologic deficits. The patients’ general conditions were evaluated according to the Karnofsky Performance Status (KPS). The neurological status was assessed based on Frankel scale. Overall survival (OS) was defined as the interval between the first diagnosis and death or the date of last follow-up. The study was terminated in December 2018, when more than one third of patients died in the validation group.

### cfDNA Extraction and Whole-Genome Low-Coverage Sequencing

Total genomic DNA and cfDNA were isolated from plasma using the Amp Genomic DNA Kit (TIANGEN) and QIAseq cfDNA Extraction kit (Qiagen), respectively. Next-generation sequencing was performed as previously described (Stover et al., 2018). cfDNA (10 ng) was used for preparation of sequencing libraries (NEBnext Ultra II). Barcoded sequencing adaptors (8 bp) were then ligated with DNA fragments and amplified by PCR. Purified sequencing libraries were massively parallel sequenced by Illumina NovaSeq 6000 platform. 4G sequencing of raw data per sample was filtered and aligned to the human reference genome.

### CIN Score in cfDNA

Plasma cell-free DNA was extracted and analyzed by Illumina NovaSeq 6000 platform. At least 10M paired reads were collected for each sample. The reads were mapped to human reference genome hg19. Genomic coverage was then counted by using software samtools mpileup (Ji et al., 2019). The mean coverage for each 200k bin was then calculated as previously reported (Mo et al., 2020). Then 100 kb, 200 kb, and 400 kb bin size were compared in Supplementary Figure S3. Z-scores for each bin were then normalized by Z-score by using the following formula:

$$Coverage_{normalized}=\frac{coverege_{raw}-mean\left(coverage_{controls,\;raw}\right)}{stdev\left(coverage_{controls,\;raw}\right)}$$ (1)

Circular binary segmentation (CBS) algorithm from R package DNA Copy (Oellerich et al., 2017) was then used to find significant genomic breakpoints and copy number changed genomics segments.

The Overall Chromosomal Instability Scores Are Summarized as

$$CIN\_score=\text{log}2\left(\underset{s\in segments}{\sum }V_{s}\times L_{s}\right)$$

where $V_{s}$ is the value of the segment and, $L_{s}\;$ is the length of the segments in unit of 200,000 base pairs.

### Statistical Analysis

Statistical analysis was performed by R project. Continuous variables are expressed as the mean ± SD. Categorical variables were compared using the Fisher exact test. The Kaplan–Meier plots were generated to estimate survival and compared using the log-rank test, using packHV package. The median follow-up was estimated by reverse Kaplan–Meier method. Univariate and multivariable Cox proportional hazards models were calculated using the survival package. The receiver operating characteristic curve (ROC) was constructed, and the area under ROC (AUC) was calculated using the ROCR package.

Raw sequencing data reported in this paper have been deposited in the Genome Sequence Archive in the BIG Data Center, Chinese Academy of Sciences that are accessible at http://bigd.big.ac.cn/gsa (ref: Database resources of the BIG Data Center in 2018). The BioProject number is PRJCA006730.

## Results

### Patient Characteristics

In the discovery group, 67 plasma samples were identified for low-coverage whole-genome sequencing. The control data acquired from healthy Chinese blood donors were generously provided by Suzhou Hongyuan Biotech Inc. (Suzhou, China). The 213 plasma samples from the validation group were used for final analysis. The mean age of the patients in the discovery and validation groups was 57.81 ± 11.33 (median 58, range 27–80) and 57.09 ± 12.20 (median 58, range 21–85) years, respectively. The median follow-up time in the two groups was 1,002 (95% CI 984–1020) and 315 (95% CI 234–396) days, with 45 (67.2%) patients and 79 (37.1%) patients passing away before the last follow-up. Differences in baseline data between the two groups were compared (Table 1).

**TABLE 1:** Patient characterization in discovery and validation group.

Factors	Discovery (2015.11-2016.5) N=67	Validation (2016.6 -2018.09) N=213	Fisher test
Age			
≥58	42	108	NS
≤57	25	105	
Gender			
Female	17	89	0.02
Male	50	124	
Primary Site			
Lung	12	49	NS
Unidentified	22	28	<0.001
Kidney	7	31	NS
Liver, Gallbladder	9	23	NS
Thyroid	7	19	NS
Breast	3	15	NS
Prostate	1	6	NS
Urine	2	2	NS
Others	4	40	0.01
Count of vertebral metastasis			
≥3	27	87	NS
2	15	46	NS
1	25	80	NS
Visceral metastases			
Unremovable	10	43	NS
Removable	3	11	NS
No Metastases	54	159	NS
Palsy			
Complete	23	75	NS
Incomplete	36	122	NS
None	8	16	NS

### Assessment of CNVs in cfDNA

**FIGURE 2:**
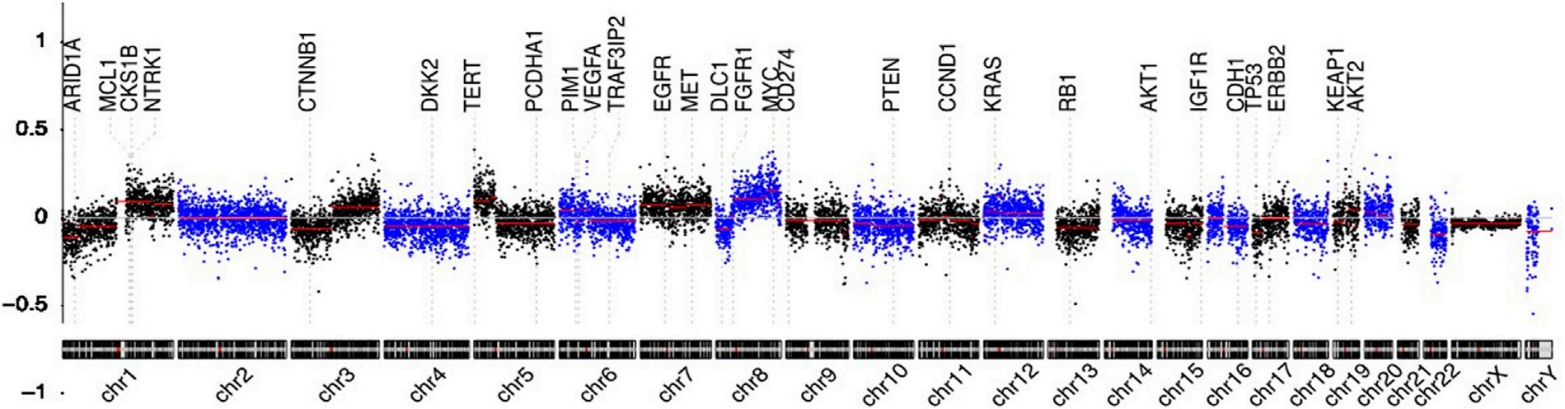
Circulating cell-free genome landscape of bone metastasis tumors. Circulating cell-free chromosomal instability of spinal metastasis cancers. Chromosomes 1, 2, … , and Y are plotted from left to right. Each dot indicates the normalized coverage value of a 200k bin. Genes of interest are marked with dashed lines.

Genome-wide overview of CNVs in 280 patients of the two groups are summarized in Figure 2. Chromosome arms 1q, 6p, 7, 8q, 20, 10p, 5p15.33, 10cen, 15q, 17q, 19, 11q13.3, and 22q were found with statistically significant copy number gains in 78.26, 47.83, 34.78, 91.30, 52.17, 30.43, 39.13, 21.74, 17.39, 39.13, 8.70, 13.04, and 13.04% samples, respectively, where well-studied oncogenes, MYC(8q), MCL1(1q), and VEGFA(6p), were located. Chromosome arms 1p, chr4, 6q, 10q, 13q, 8p, 11q, 11p, chr16, chr9, 17p, 21q, 14q, chr3, chr18, and 8p were found with statistically significant copy loss in 65.22, 60.87, 47.83, 60.87, 65.22, 52.17, 39.13, 43.48, 43.48, 47.83, 52.17, 30.43, 43.48, 21.74, 26.09, and 34.78% of samples, respectively, where potential tumor suppressor genes DLC1(8p), DKK2(4q), PTEN(10q), and TP53(17p) were located.

Detectable chromosomal changes were much less pronounced in thyroid and kidney cancer than those in lung, liver, breast, and head and neck cancers. For tumors with high CNVs, chromosome 1q gain and 8q gain were common. Chr6p gain and 4q loss were the most frequent in cancers originating from the liver. 3q gain was the most frequent in lung, head and neck, and breast cancers. Chr7 gain was the most in lung cancer and head and neck cancer. Chr20 gain was detectable in liver and breast cancers.

We also ran our data through ichorCNA algorithm (https://github.com/broadinstitute/ichorCNA) and found a linear relationship of tumor fraction between our pipeline and ichorCNA pipeline. ctDNA% also showed a positive correlation with the estimated CIN score in this study (Supplementary Figure S2).

### Increased CNVs Predict Worse Survival

**FIGURE 3:**
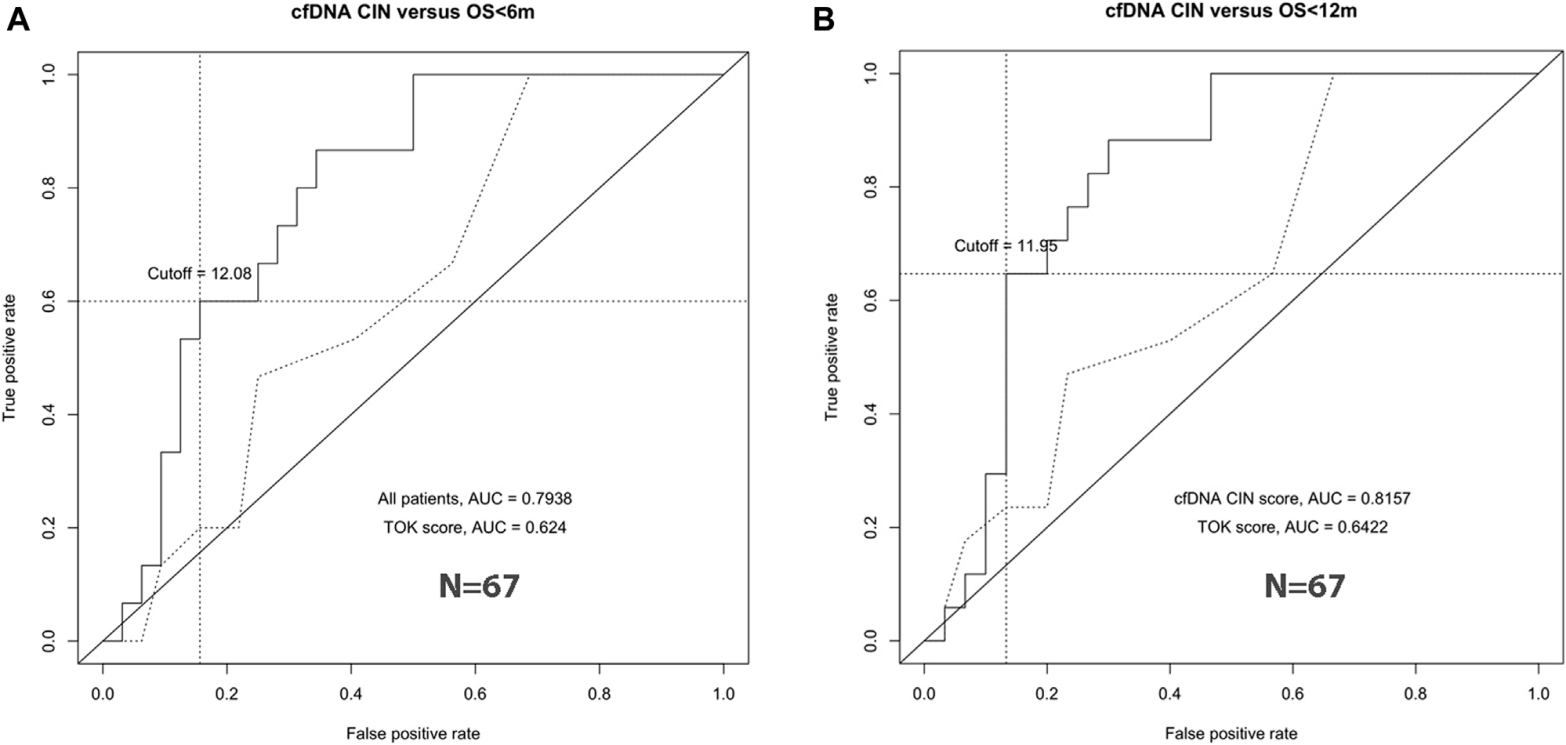
CIN cutoff by ROC curve analyses. The best cutoff to predict 6-months survival **(left, A)** and 12-months survival **(right, B)**.

In the discovery group, survival ROC curves were generated to identify the specific threshold of the CIN score for the classification of long-survival patients and short-survival patients. For 6- and 12-months survival prediction, the AUC value was 0.7938 (95% CI 0.6657–0.9218) and 0.8157 (95% CI 0.6922–0.9392) using CIN score, with the threshold of 12.08 and 11.95 (Figures 3A,B). Based on the results in the discovery group, patients with CIN score ≥12.0 were categorized as high-CIN status and those with a lower score as low-CIN status. The median OS of patients with high- or low-CIN status was 213 (95% CI 66.2–360) and 810 (95% CI 369–1251) days, respectively.

**FIGURE 4:**
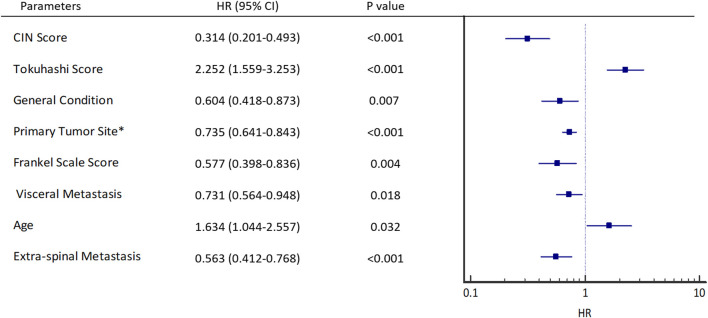
Univariant hazard ratios. *Primary site of cancer according to Tokuhashi score, 5 (Thyroid, Prostate, Breast), 4 (Rectum), 3 (Kidney, Uterus), 2 (Others), 1 (Liver, Gallbladder, Unidentified), 0 (Lung, Osteosarcoma, Stomach). General condition is estimated by KPS score, KPS (Good: 80–100, Moderate: 50–70, Poor: 10–40).

In the validation group, univariate analysis showed that patients with cfDNA CIN score <12 (HR 0.314, 95% CI 0.201–0.493, *p* < 0.001), Tokuhashi score, > 8 (HR 2.252, 95% CI 1.559–3.253, *p* < 0.001), better general condition (HR 0.604, 95% CI 0.418–0.873, *p* = 0.007), low malignant primary tumor (HR 0.735, 95% CI 0.641–0.843, *p* < 0.001), a better Frankel scale score (HR 0.577, 95% CI 0.398–0.836, *p* = 0.004), inoperable visceral metastasis (HR 0.731, 95% CI 0.564–0.948, *p* = 0.018), age ≥58 (HR 1.634, 95% CI 1.044–2.557, *p* = 0.032), and extra-spinal metastasis (HR 0.563, 95% CI 0.412–0.768, *p* < 0.001) were statistically significant for short survival (Figure 4).

Furthermore, cfDNA CIN score <12 (Table 2, HR 0.561, 95% CI 0.447–0.703, *p* < 0.001) and Tokuhashi score, ≤ 8 (Table 2, HR 2.258, 95% CI 1.561–3.266, *p* < 0.001) both reached statistical significance by two-parameter Cox regression analysis. Also, all these eight variables were used for multivariate analysis, finding that the primary tumor site (HR 0.728, 95% CI 0.620–0.855, *p* < 0.001), Tokuhashi score, ≤ 8 (HR 2.886, 95% CI 1.318–6.323, *p* = 0.008), extra-spinal metastasis (HR 0.529, 95% CI 0.384–0.729, *p* < 0.001), and cfDNA CIN score <12 (HR 0.322, 95% CI 0.204–0.507, *p* < 0.001) were predictors of survival for patients with spinal metastasis (Table 3).

**TABLE 2:** Two-parameter Cox-regression analysis.

	P	HR	95% CI
cfDNA CIN_score < 12	<0.001	0.561	0.447–0.703
TOK_Score ≤ 8	<0.001	2.258	1.561–3.266

**TABLE 3:** Eight-parameter multivariate survival analyses.

	P	HR	95% CI
cfDNA CIN_score < 12	<0.001	0.322	0.204–0.507
Primary site	<0.001	0.728	0.620–0.855
Tokuhashi score ≤ 8	0.008	2.886	1.318–6.323
Ex- vertebral bone metastases	<0.001	0.529	0.384–0.729

### Discrimination of Long-Survival Patients With Poor Tokuhashi Score

**FIGURE 5:**
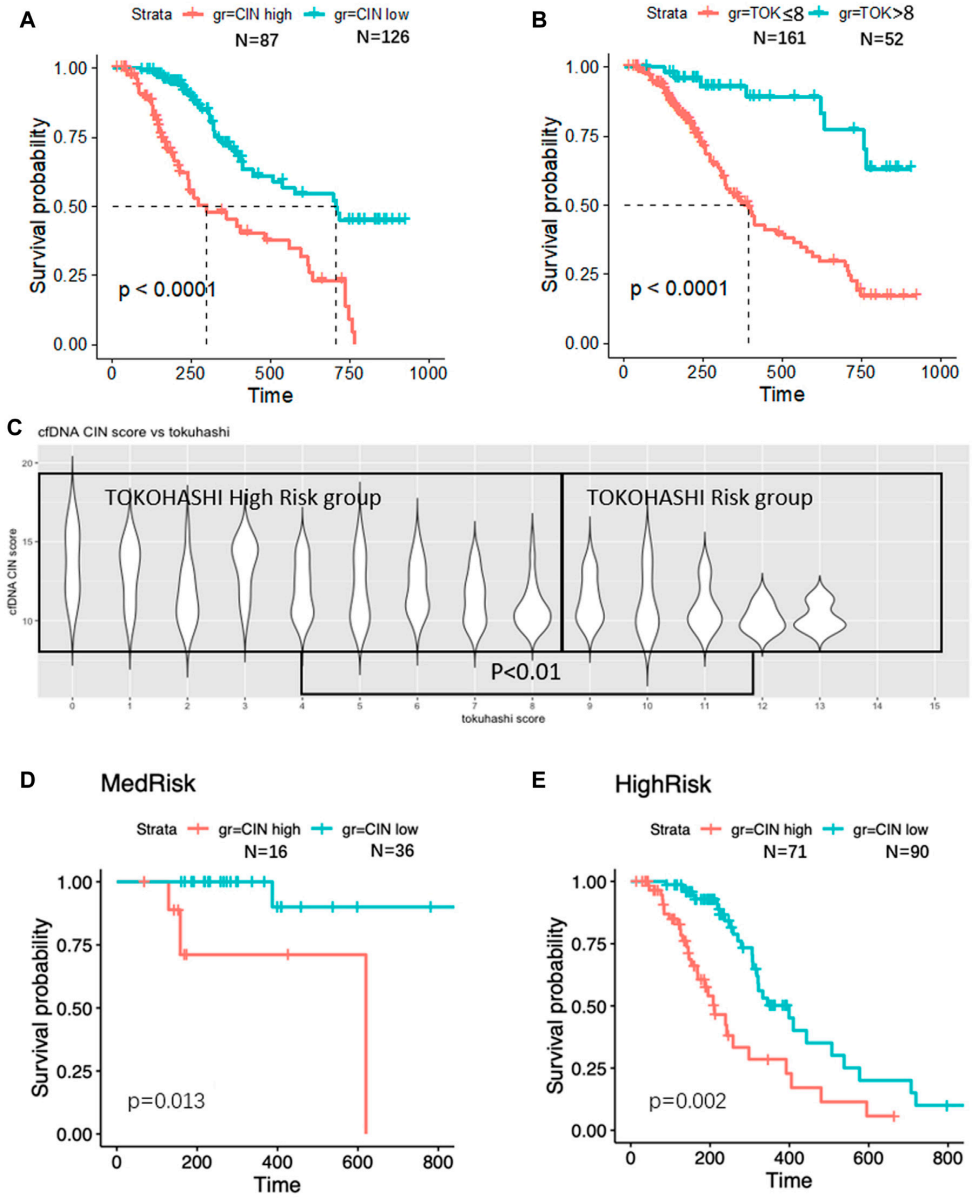
Survival analyses. Significantly worse survival was found in CIN high **(A)** and low TOK score **(B)** patients. CIN is higher in TOK high-risk group **(C)**. In TOK medium-risk group, higher CIN was found associated with poor survival **(D)**. In TOK high-risk group, higher CIN was found associated with poor survival **(E)**.

Knowing that Tokuhashi score is widely used in prognostic prediction of spinal metastasis patients, we tested its accuracy in the validation group (Figure 5B) and found that 161 patients with low Tokuhashi score (≤8) in the validation group had significantly shorter median OS (392 days, 95% CI 313–471), as compared with the median OS of the other 52 patients with Tokuhashi score >8 (median survival unreached, HR 2.252, 95% CI 1.559–3.253, *p* < 0.001). The median OS was 298 days (95% CI 129–467) for patients with high-CIN scores versus 707 days (95% CI 501–913) for patients with low-CIN scores (HR 0.314, 95% CI 0.201–0.493, *p* < 0.001, Figure 5A). Distribution of CIN score on each Tokuhashi score is shown in Figure 5C. Patients with high Tokuhashi score had lower CIN score (*p* < 0.01).

However, 99 (61.5%) of the 161 patients with low Tokuhashi score had predictive survival of less than 6 months, while their actual survival was more than 6 months. Next, in the low Tokuhashi score group, we tested whether the CNV status could discriminate the good-prognosis patients from the poor-prognosis ones. The median OS was 258 days (95% CI 184–332) for patients with high-CIN scores versus 443 days (95% CI 301–585) for patients with low-CIN scores (*p* = 0.002, Figure 5E). With the help of the CIN score, spinal metastasis patients with low Tokuhashi score could be divided into two different subgroups in terms of survival. Similarly, the median OS in the high Tokuhashi score group was also longer in the low-CIN patients than that in the high CIN patients (*p* = 0.013, Figure 5D).

### The Performance of Combined use of Tokuhashi and cfDNA CIN Scores in Predicting Short- and Long-Survival Patients

We then combined Tokuhashi and CIN scores as a predictor of patient OS. As shown in Table 4, CIN scores larger than 12 defined the worst survival group of 87 (40.8%) patients, of whom 50 patients (57.5%) died within 6 months. The 90 (42.3%) patients with Tokuhashi scores ≤8 and CIN scores less than 12 showed a better prognosis, with the 6-months death rate of 22.2%. The other 36 patients with Tokuhashi scores >8 and CIN scores less than 12 showed the best survival, with the 6-months death rate of 5.56%, and 18 (50.0%) of them showed OS longer than 12 months.

**TABLE 4:** The performance of predicting of short and long-survival patients by combining Tokuhashi score and cfDNA CIN scores.

		<6 months	6–12 months	≥12 months
High risk (N=87)	UCAD≥12	50 (57.5%)	18 (20.7%)	19(21.8%)
Median risk (N=90)	TOK≤8, UCAD<12	20 (22.2%)	43 (47.8%)	27 (30.0%)
Low risk (N=36)	TOK>8, UCAD<12	2 (5.56%)	16 (44.4%)	18 (50.0%)

## Discussion

Spinal metastasis is a common occurrence in multiple advanced cancers (Bartels et al., 2008). Pretreatment survival prediction may help clinicians in the decision-making process for subsequent treatments. Several survival predictive models have been developed over the past decades, but their accuracy is unsatisfactory (Tan et al., 2016). On the other hand, there is a lack of real-time biomarkers with consideration of the molecular characteristics of cancer cells for survival prediction (Bauer et al., 2002). It was found in this study that CNVs from cfDNA could act as a potential biomarker for prognosis prediction of patients with spinal metastasis and help discriminate long-survival patients from poor-prognosis patients assessed by traditional prediction models in the perspective of molecular characteristics of malignancies.

Copy number variation is a hallmark in various cancers, including metastatic cancers, and deemed a prognostic predictor associated with an increased risk of recurrence or death (Gronroos and López-García, 2018; Tan et al., 2019). In this study, cfDNA CNV was first proved to be a potential biomarker for predicting the survival of patients with spinal metastasis. Vanderstichele et al. (Vanderstichele et al., 2017) reported that evaluating the cfDNA with CNV could help distinguish the patients with an adnexal mass caused by an invasive carcinoma from patients presenting with a benign adnexal mass. In addition, more genetic and epigenetic alterations could also be detected by cfDNA sequencing (Ellinger et al., 2011). Mehra et al. (2018) demonstrated that the cfDNA fragment concentration was an independent prognostic variable for radiological progression-free survival and OS of patients with metastatic prostate cancer. Stover et al. (2018) reported that tumor fraction was associated with worse survival in metastatic triple-negative breast cancer patients, and specific somatic copy number alterations were enriched in these patients. Spinal metastases originating from multiple types of cancer are featured by heterogeneous molecular characters, which impose a significant challenge on clinicians in precise diagnosis, evaluation, and treatment (Jansson and Bauer, 2006; Kawahara et al., 2009). As aforementioned, CNV could present the common properties of metastatic malignancies. Our results demonstrated that CNV in cfDNA was significantly associated with the survival of patients with spinal metastasis, which provides a universal and convenient tool of pretreatment evaluation for different cancers as an integrated entity. This interpretation of CNV could be applicable to the surveillance of progression of malignancies in other scenarios.

Another intriguing finding of our study is that CNV successfully discriminated the good-prognosis patients with low Tokuhashi score from poor-prognosis patients. According to the revised Tokuhashi score, the predicted survival of patients with a total score ≤8 was less than 6 months (Tokuhashi et al., 2005). However, it was found that Tokuhashi score might not be qualified to establish a precise prediction of survival, especially in predicting short-term survival (Ahmed et al., 2018). Some patients ranked with a low Tokuhashi score may possess a relatively longer survival than expected. Ahmed et al. (2018) reported that the revised Tokuhashi score was not accurate in 90-days survival prediction after surgery, with the AUC being 0.67. The data of Tan et al. (2016) suggested that only 41.7% patients had a Tokuhashi-predicted survival that was correlated with actual survival. Judged by Tokuhashi score, only 6.1% patients were predicted to survive more than 6 months, while 44.4% of patients in the cohort attained survival longer than half a year. Moreover, the mean OS of patients with spinal metastases have been observed to be improved and longer than 1 year in the recent literature (Tang et al., 2015; Yang et al., 2019), which is in congruence with our data. As CNV level can reflect the disease progression through the actual activity of the tumor individually, it proved capable of predicting the prognosis of patients with highly malignant spinal metastases in the current study, including lung cancer. Identification of the patients who have long-term survival in the low Tokuhashi score group can contribute to a more positive alteration of treatment, thus improving their actual prognosis. Pelegrini de Almeida et al. (2018) retrospectively analyzed 117 patients and found that the patients with low Tokuhashi score for whom surgical treatment was not recommended traditionally had better quality of life and longer survival after adequate surgery than the results inferred by the Tokuhashi system. It was found in our study that patients with poor Tokuhashi score but low CNV had relatively good prognosis, with the median OS of 433 days. Accurate survival prediction of this part of patients through cfDNA test might optimize the selection of their treatment procedures.

Unlike the traditional predictive models for metastatic malignancies such as the Tokuhashi score, which may be influenced by subjective confounders, CNV is calculated based on the plasma cfDNA, which represents a more objective set of criteria. Almost all previous models focus on clinical characteristics of the patients with spinal metastases, while CNV reflects the tumor status of progression in circulation (Batista et al., 2016; Vanderstichele et al., 2017). Although other blood tests including certain tumor biomarkers (CEA, AFP, etc.), inflammation-based biomarkers (platelet–lymphocyte ratio, neutrophil–lymphocyte ratio, etc.), and hemostasis biomarkers (D-dimer, fibrinogen, etc.) were also reported to be associated with survival of cancer patients, they are either specific only for a single tumor type or lack sensitivity (Ay et al., 2012; Li et al., 2018; Yang et al., 2019). Examining CNV in cfDNA for prognosis prediction provides more information on the innate and dynamic character of metastatic malignancies, which are often ignored in most traditional models. Furthermore, accurate survival prediction through CNV may help make a more precise treatment decision. It has been deemed as the principle that the choice of treatment should be judged by the prognosis of spinal metastatic patients (Tokuhashi et al., 1990; Tomita et al., 2001). For good-prognosis patients, radical excision with wide or marginal margin is suggested for long-term local control, while for patients with poor prognostic prediction, intralesional excision or palliative treatment can reduce unnecessary iatrogenic injury (Tomita et al., 2001).

The main limitation of this study is that all blood samples were obtained from the cohort in one single institution. The patients recruited in our study are relatively homogeneous, and were all diagnosed with spinal metastases, implying that our conclusion should be interpreted cautiously in patients with metastatic sites involving other systems. Besides, we noticed different features of CIN and different clinical outcomes between tumors from various origins, but the score was worse when estimating each carcinoma (Supplementary Figure S1) than that of pan-cancer as a whole (Supplementary Figure S1). Thereby, an increased number of patients and further investigation taking into account the differences between malignancies are required to improve the performance of the algorithm. Second, as the cohorts of the discovery group and validation group were not evaluated synchronously, relevant biases may not be avoidable. The constitution of the malignant types is somewhat different between the two groups; for instance, fewer patients in the validation group were diagnosed with cancers of unknown primary sites than those in the discovery group (*p* < 0.001). Third, clinical advances in imaging and histopathological examinations with specific immunohistochemistry in more recent years may also affect tumor diagnosis and increase the sensitivity of primary tumor detection (Collado Martín et al., 2014; Varadhachary and Raber, 2014). Finally, more female patients were included in the validation group than those in the discovery group (*p* = 0.02).

In summary, we proposed a framework for the minimally invasive examination of genetic architecture of spinal metastatic cancers. The interpretation of its result was proved to be correlated with the clinical outcome of patients. A high cfDNA CIN score suggests a dismal survival of patients with spinal metastasis. cfDNA testing for CNV could be applied into clinical practice as a new indicator for the prognosis of spinal metastasis. Accurate selection of patients with either predicted long or short survival might improve the treatment planning and ameliorate the clinical outcome.

## Key points

A CIN score as a new quantified survival indicator is developed for survival prediction of patients with spine metastasis.

## Importance of the study

The skeletal system is the third most common metastatic site of most cancers. About 19.7% of the terminal-stage cancer patients with metastasis diagnosed during 2015–2018 survived more than 2 years, and over half of them were still alive until the last follow-up in our center. However, the widely used revised Tokuhashi score lacks accuracy in prognostic prediction. About 50% of the patients with a Tokuhashi score under 8 survived for more than 1 year but were tortured by both physical suffering and mental desperation. In this study, we present a CIN score as a new quantified indicator for the survival prediction of such patients. The CIN score is a more objective set of criteria that can reflect both molecular characteristics of all cancer subclones and tumor burden instead of traditional symptoms and clinically oriented scores. Therefore, it can ensure the optimal therapeutic efficiency by helping make a more precise clinical decision.

## Data Availability

The datasets presented in this study can be found in online repositories. The names of the repository/repositories and accession number(s) can be found below: National Genomics Data Center under BioProject number PRJCA006730.

## Supplementary Material

Supplementary Figure S1 The survival curve of different carcinomas. The prognosis was compared between high CIN status and low CIN status in each carcinoma.

Supplementary Figure S2
**(A)** The comparison of tumor fraction estimation performed by our method and ichorCNA algorithm. **(B)** The correlation of ctDNA% and the estimated CIN score.

Supplementary Figure S3
**(A)** The correlation of 100 kb bin size and 200 kb bin size. **(B)** The correlation of 400 kb bin size and 200 kb bin size.

## Abbreviations

AFP: alpha fetoprotein; AUC, area under ROC; bp, base pair; CEA, carcinoembryonic antigen; cen, centromere; cfDNA, Cell-free DNA; chr, chromosome; CIN, chromosome instability; CNV, copy number variation; DKK, dickkopf; DLC, deleted in liver cancer; DNA, deoxyribonucleic acid; EDTA, ethylenediaminetetraacetate; KPS, Karnofsky Performance Status; MCL, mantle cell lymphoma; ng, nanogram; NS, not significant; OS, overall survival; p, petit; PTEN, phosphatase and tensin homolog; ROC, operating characteristic curve; TOK, Tokuhashi score; TP53, tumor protein 53; VEGFA, vascular endothelial cell growth factor A.
