# Supplementary figures and images for: Quantified CIN Score From Cell-free DNA as a Novel Noninvasive Predictor of Survival in Patients With Spinal Metastasis
Source: Front Cell Dev Biol. 2021 Dec 9;9:767340. doi: 10.3389/fcell.2021.767340 (PMC8696126; doi:10.3389/fcell.2021.767340)

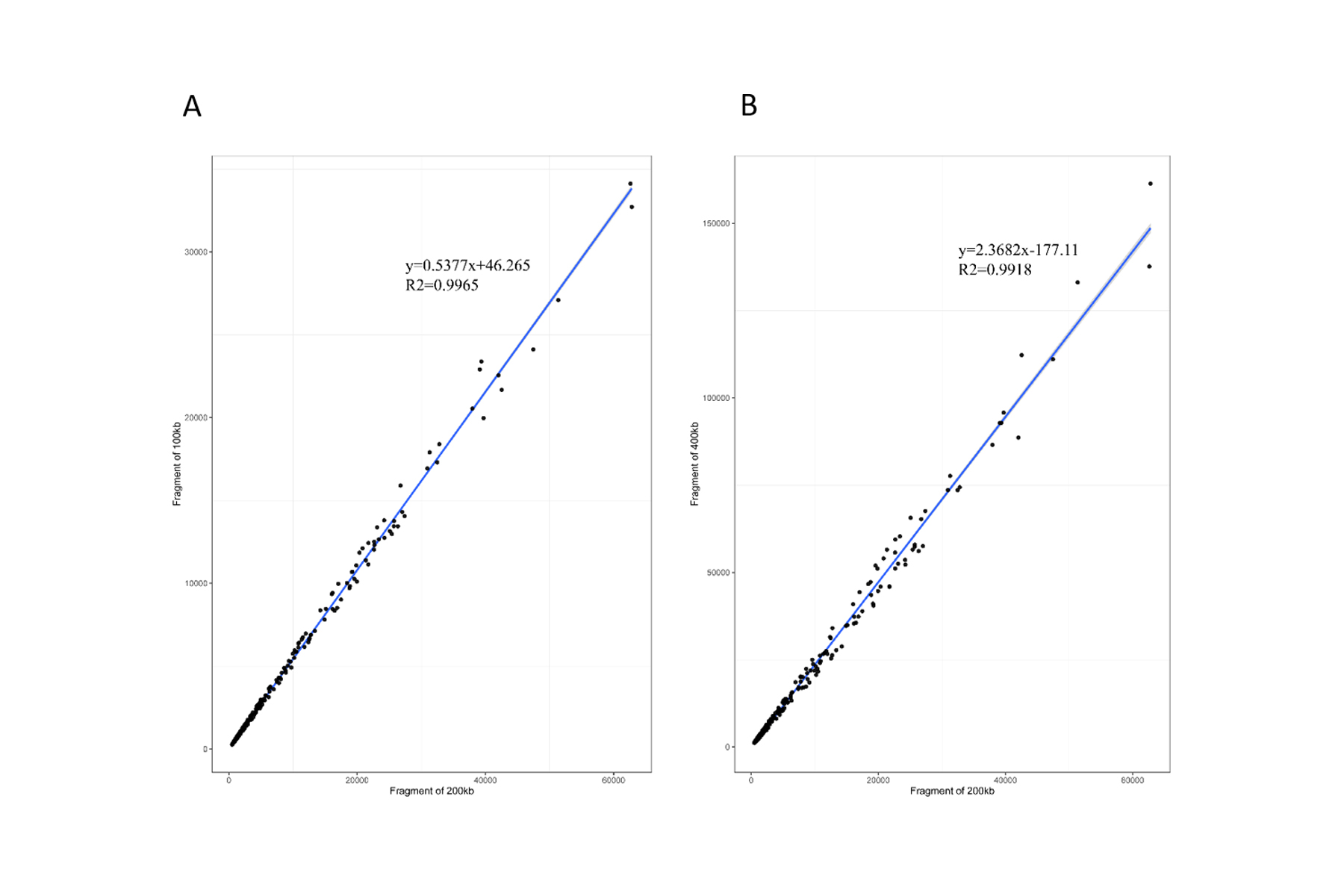

Supplement: Supplementary file 1 [file Image3.JPEG]

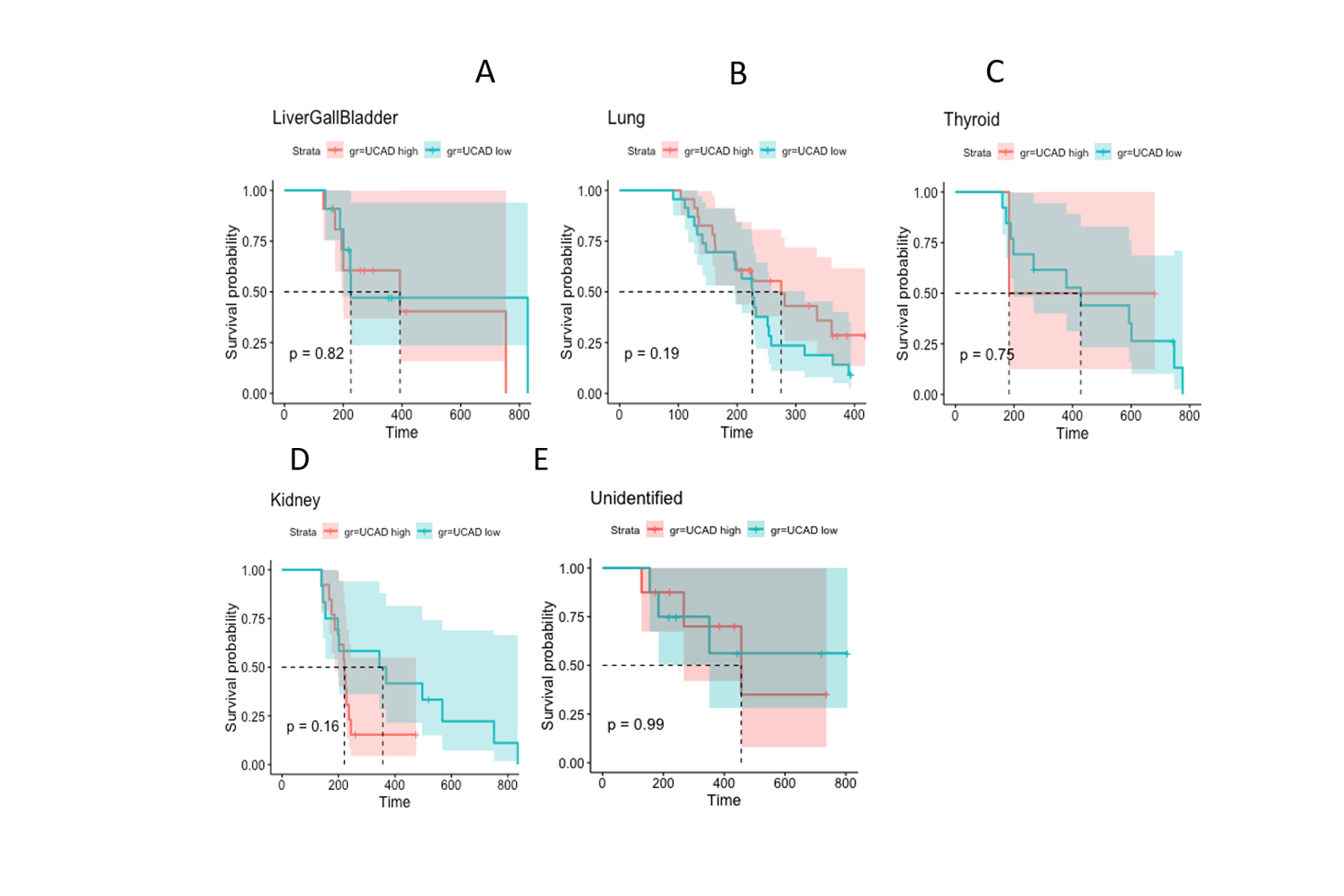

Supplement: Supplementary file 2 [file Image1.JPEG]

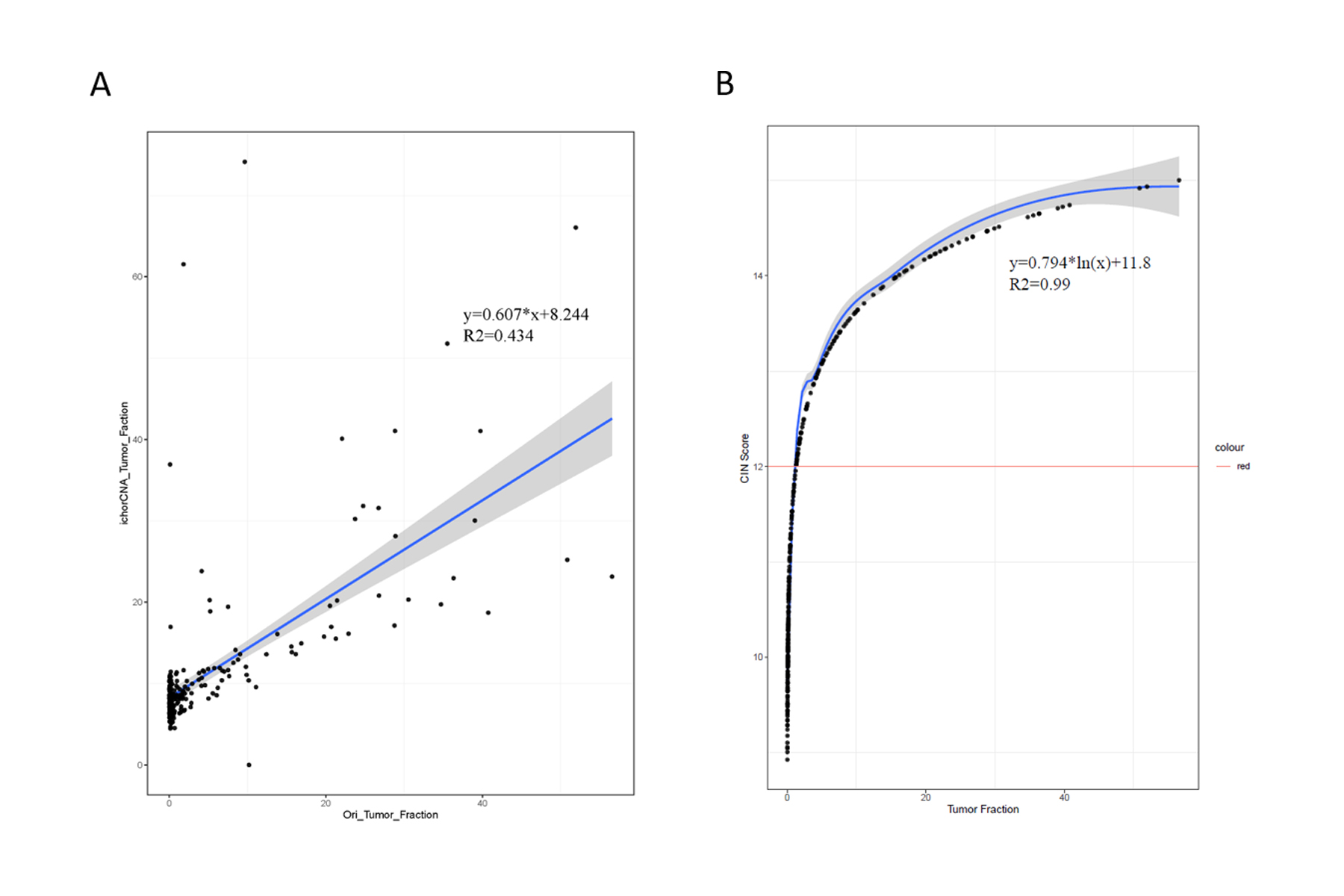

Supplement: Supplementary file 3 [file Image2.JPEG]
